# Disentangling the role of users’ preferences and impulsivity traits in problematic Facebook use

**DOI:** 10.1371/journal.pone.0201971

**Published:** 2018-09-05

**Authors:** Stephane Rothen, Jean-François Briefer, Jory Deleuze, Laurent Karila, Cecilie Schou Andreassen, Sophia Achab, Gabriel Thorens, Yasser Khazaal, Daniele Zullino, Joel Billieux

**Affiliations:** 1 Addiction Division, Department of Mental Health and Psychiatry, University Hospitals of Geneva, Geneva, Switzerland; 2 Research Center for Statistics, Geneva School of Economics and Management, University of Geneva, Geneva, Switzerland; 3 Laboratory for Experimental Psychopathology, Psychological Sciences Research Institute, Université Catholique de Louvain, Louvain-La-Neuve, Belgium; 4 Service d'Addictologie, Hôpital Universitaire Paul Brousse, AP-HP, Université Paris Sud, INSERM U1000, Villejuif France; 5 Department of Clinical Psychology, University of Bergen, Bergen, Norway; 6 Faculty of Medicine, University of Geneva, Geneva, Switzerland; 7 Addictive and Compulsive Behaviours Lab (ACB-Lab), Institute for Health and Behaviour, University of Luxembourg, Esch-sur-Alzette, Luxembourg; University of Padua, Italy

## Abstract

The use of social network sites (SNSs) has grown dramatically. Numerous studies have shown that SNS users may suffer from excessive use, associated with addictive-like symptoms. With a focus on the popular SNS Facebook (FB), our aims in the current study were twofold: First, to explore the heterogeneity of FB usage and determine which kind of FB activity predicts problematic usage; second, to test whether specific impulsivity facets predict problematic use of FB. To this end, a sample of FB users (*N* = 676) completed an online survey assessing usage preferences (e.g., types of activities performed), symptoms of problematic FB use and impulsivity traits. Results indicated that specific usage preferences (updating one’s status, gaming via FB, and using notifications) and impulsive traits (positive and negative urgency, lack of perseverance) are associated to problematic FB use. This study underscores that labels such as FB “addiction” are misleading and that focusing on the actual activities performed on SNSs is crucial when considering dysfunctional usage. Furthermore, this study clarified the role of impulsivity in problematic FB use by building on a theoretically driven model of impulsivity that assumes its multidimensional nature. The current findings have identifiable theoretical and public health implications.

## Introduction

Over the last decade, online social network sites (SNSs) have grown dramatically. For instance, in April 2018, Facebook (FB), one of the most well-known SNSs, had 2.20 billion active monthly users (https://zephoria.com/top-15-valuable-facebook-statistics/). The success of SNSs can be viewed as being closely tied to the inherent human need to belong to communities and to affiliate with peers to engage in a role within the community and obtain the social acceptance and gratification that comes with its fulfillment. Their success also relates to the need for self-presentation, which is part of the process of impression management [1].

FB use (i.e., time spent on FB) seems to be associated with offline social capital, as suggested by some studies. For instance, a cross-sectional study highlighted a positive link between FB use and offline connections, i.e. relationships in real life [2]. Similarly, a longitudinal study showed that regular FB users tend to benefit from a rich offline social network [3]. Perceived social isolation seems, however, to increase with high involvement in social media use (including FB), as found in a recent study on representative US individuals aged 19–32 years [4]. Yet, because this was a cross-sectional study, causality cannot be established. On the one hand, social isolation can lead to increased involvement in SNS as an emotional coping strategy or as a strategy to search for social support; on the other hand, overuse of SNS per se can induce social isolation (e.g., a sense of exclusion from online social groups, self-negative comparison with the idealized life of online peers, decreased available time for offline social relationships). Furthermore, a Dutch study conducted on 10- to 19-year-old adolescents [5] showed that receiving positive feedback on SNS profiles increased self-esteem and well-being, whereas receiving negative feedback on SNS profiles had the opposite effect. Another study [6] highlighted that self-management of SNSs positively influenced self-esteem. More precisely, these authors found that participants who were instructed to update and view their own profile during an experiment reported a higher level of self-esteem.

Despite ongoing controversies regarding the status of excessive SNS use as a potential addictive disorder [7], a growing number of research articles have reported that SNS users may suffer from excessive and potentially impairing use [8]. For instance, overinvolvement in SNSs has been linked to addiction-like symptoms [9] and a variety of negative psychological correlates or consequences [10–13]. Some studies have also tried to disentangle the psychological factors involved in problematic use of SNS, with, on the one hand, a focus on user preferences and motivations and, on the other, a focus on the normal and pathological personality traits that could act as risk factors. In the current paper, we decided to use the label “problematic SNS use” to define an involvement linked to negative (personal, social, professional/academic) consequences, in order to avoid *a priori* considering it as a genuine addictive disorder and allows for potential alternative conceptualizations [14]. For example, some studies showed that excessive SNS use can be viewed as a maladaptive coping [15], and assimilating it to a disorder might result in (over)pathologization [16].

SNSs, and FB in particular, can be used in different ways and can fulfill various motives [3], including, but not limited to, entertainment, social sharing, information seeking, relationship maintenance (e.g., interacting with an existing offline social network), and emotional coping (e.g., facing boredom, loneliness, or negative affect; [17]. In particular, FB usage preferences (and underlying motives) have been related to its healthy versus dysfunctional use [8,18–20]. Directed communication, for instance (e.g., one-on-one exchanges), was found to have more impact on social capital than does broadcasting or passive consumption of social news [3]. Moreover, Baek, Bae, & Jang [21] emphasized the opposite effects of social (i.e., bidirectional, such as chatting or messaging) versus para-social (i.e., unidirectional, such as checking other’s status or profile) online activities: Social activities were negatively correlated with a perceived feeling of loneliness, whereas para-social activities were positively correlated with it. In another study, well-being was found to increase when time spent on FB was used to maintain relationships, but to decrease when it was used to create new relationships [18].

In recent years, a growing number of studies have explored the normal and pathological personality traits likely to promote problematic use of SNSs, in particular FB [for a review, see 8]. The initial studies were conducted by using the five-factor model (FFM) of personality [22] and on the whole showed that problematic SNS use is associated with higher neuroticism and extraversion and with lower conscientiousness[9,23–24]. Symptoms of SNS addiction were also found to be positively related to lower levels of self-esteem [25,26] and insecure attachment [27]. Problematic and addictive-like use of SNSs has also been linked to pathological personality traits, namely, proneness to narcissistic personality [28], obsessive-compulsive symptoms [10], and borderline personality traits [29].

Strikingly, existing data on the role of impulsive personality traits in problematic use of SNSs remain scarce, although a few reports have documented heightened impulsivity in individuals displaying such use [30]. This is particularly surprising, given the tremendous number of studies documenting the associations between impulsivity and a wide range of addictive behaviors [31], including general or specific types of Internet-related problematic behaviors such as problematic video gaming [32–34]. Despite recent models of Internet-related disorders underscoring the pivotal role of impulsivity traits in the disorder [35]), the available data regarding problematic use of SNSs are to date limited.

An important gap in the literature is the exploration of the links between impulsivity and problematic SNS use built upon a theoretically driven model of impulsivity that assumes its multidimensional nature. In this regard, the UPPS-P (Urgency-Premeditation-Perseverance-Sensation seeking-Positive urgency) model of impulsivity [36,37] measures five impulsivity facets: (1) negative urgency, defined as the tendency to act rashly while faced with intense negative emotional contexts; (2) premeditation, defined as the tendency to take into account the consequences of an act before engaging in that act; (3) perseverance, defined as the ability to remain focused on a task that may be boring and/or difficult; (4) sensation seeking, considered as the tendency to enjoy and pursue activities that are exciting and openness to trying new experiences; and (5) positive urgency, defined as the tendency to act rashly while faced with intense positive emotional contexts. In the last decade, this model has proven to be a relevant theoretical framework to elucidate the associations between specific impulsivity traits and various forms of psychiatric disorders and problematic behaviors and thus became a dominant model in the field of psychopathology [38] and neuropsychology [39]. However, to date, the UPPS model has not been used to explore the links between specific impulsivity facets and problematic use of SNSs.

Accordingly, the aims of the current study were twofold. The first objective was to explore the heterogeneity of FB usage preferences in a community sample and to determine which type of FB activity puts people more at risk for developing problematic usage. The second objective was to test whether specific impulsivity facets predict problematic use of SNSs by capitalizing on their multidimensional nature. Our study is of an exploratory nature and thus formal operational hypotheses were not formulated. Yet, it can be expected that problematic FB use will be associated to elevated levels of positive and negative urgency, as previous research identified ICT-mediated problematic behaviors (e.g., dysfunctional use of online sexual activities, sexting) to be predicted by these specific impulsivity components [40,41]. If the current study reproduces these findings, it will suggest that FB problematic use constitutes an unregulated form of behavior displayed to regulate emotional states in the short term, despite the potential delayed negative consequences.

## Methods

### Participants and procedure

The study consisted of a survey that was accessible online and circulated via FB and other social networks and research networks, as well as via email by using snowballing techniques among the researchers’ contacts. The survey was disseminated by using an online platform (Qualtrics, Provo, UT). All items used in the online survey can be obtained from https://osf.io/xt4zv/. The survey items were administered in fixed order (FB actual-use items, FB problematic use items, a self-esteem item, and impulsivity items; see below). The survey was available between October 1 and November 30, 2015. Anonymity of the participants was guaranteed (no personal data or Internet Protocol [IP] address was collected). Inclusion criteria were being French-speaking, aged 18 years or older, and being a FB user. The sample included 857 participants, of whom 676 fully completed the questionnaire. The mean age of the completers was 35.8 years (*SD* = 12.0, range: 18.3–80.3 years), and 466 (68.9%) were women. The median age of women (30.8 years) was lower than that of men (38.8 years; W = 60770, *p* < 0.001). The ethical committee of the Psychological Sciences Research Institute of the Catholic University of Louvain, Louvain-la-Neuve, approved the study.

### Measurements

The study included several questions generated to measure actual FB use, including frequency of use (separately for weekday and weekend), types of activities performed on FB (see Table 1), preferred activities performed on FB (participants ranked the activities from preferred to less preferred; see Table 2), device(s) used to access FB (personal computer, computer at work, smartphone, tablet), preferred device used to access FB, use of Messenger (do participants use FB alone, Messenger alone, or both applications when using FB via smartphones or tablets; this variable was coded as Messenger use: yes/no), and usage of notifications (yes/no).The frequency of use was not taken into account for further analyses, as the reliability of self-reported time spent in SNS. The different activities measured resulted from a consensus of the research team. All activities identified by the research team were considered for the study. The frequency item was finally not used in our analyses, as recent evidences demonstrated that self-reported frequency of FB is not a reliable variable [42,43].

**Table 1:** FB usage preferences and gender differences.

	Whole sample (*n* = 676)	Men (*n* = 210)	Women (*n* = 466)	χ^2^	*p*-Value
Reading the news feed *n* (%)	389 (57.5%)	137 (65.2)	252 (54.1)	6.9	>0.01
Viewing friends’ pictures *n* (%)	363(53.7)	94 (44.8)	269 (57.7)	9.3	0.1
Commenting *n* (%)	353 (52.2)	114 (54.3)	239 (51.3)	0.4	0.5
Reading friends’ timelines *n* (%)	346 (51.2)	98 (46.7)	248 (53.2)	2.3	0.1
Contributing to a group *n* (%)	235 (34.8)	70 (33.3)	165 (35.4)	0.2	0.7
Updating status *n* (%)	229 (33.9)	95 (45.2)	134 (28.8)	16.8	>0.001
Gaming *n* (%)	66 (9.8)	12 (5.7)	54 (11.6)	5.0	0.025
Sharing stuff from the Internet *n* (%)	44 (6.5)	14 (6.7)	30 (6.4)	0.0	1
Using Messenger app *n* (%)	294 (43.5%)	90 (42.9%)	204 (43.8%)	0.02	0.89
FB main access				24.08	>0.001
*Personal computer n* (%)	241 (35.7%)	99 (47.1%)	142 (30.5%)		
*Professional computer n* (%)	46 (6.8%)	19 (9.0%)	27 (5.8%)		
*Smartphone n* (%)	351 (51.9%)	81 (38.6%)	270 (57.9%)		
*Tablet n* (%)	38 (5.6%)	11 (5.2%)	27 (5.8%)		
Notifications *n* (%)	302 (44.7%)	75 (35.7%)	227 (48.7%)	9.38	>0.01

**Table 2:** Favorite FB activities and related gender differences.

	Whole sample^1^	Men	Women	Wilcoxon	*p*-Value
Reading the news feed (*n* = 389) mean (*SD*)	1.9 (1.2)	1.8 (1.1)	1.9 (1.2)	17432	0.9
Reading friends’ timelines (*n* = 346) mean (*SD*)	2.2 (1.3)	2.4 (1.3)	2.2 (1.3)	13256	0.2
Updating status (*n* = 229) mean (*SD*)	2.6 (1.4)	2.2 (1.2)	2.8 (1.4)	4694	0.0005
Commenting (*n* = 353) mean (*SD*)	2.7 (1.1)	2.6 (1.0)	2.8 (1.1)	12422	0.2
Contributing to a group (*n* = 235) mean (*SD*)	2.7 (1.5)	2.8 (1.5)	2.6 (1.5)	6370	0.2
Sharing stuff from the Internet (*n* = 44) mean (*SD*)	2.8 (1.7)	3.4 (1.9)	2.5 (1.6)	272	0.1
Viewing friends’ pictures (*n* = 363) mean (*SD*)	2.9 (1.4)	3.1 (1.4)	2.8 (1.3)	14123	0.08
Gaming (*n* = 66) mean (*SD*)	2.9 (1.7)	1.9 (1.0)	3.1 (1.8)	198	0.032

^1^These scores are ranks, meaning that lower scores indicate higher preferences.

Problematic use of FB was measured with a revised version of the Internet Addiction Test (IAT-R). The IAT-R is a modified version of the IAT-20 Young questionnaire on Internet addiction. It has been modified for statistical reasons in order to take into account current Internet use and the fact some items of the original version are outdated [44, 45], and to include a question on craving, which was not measured in the original version of the IAT. Indeed, previous research emphasized the relevance of using a craving item when assessing problematic and addictive-like online activities [46]. The IAT-R comprises 18 items scored on a 5-point Likert scale ranging from 1 to 5. Cronbach’s alpha for the entire scale, computed on the polychoric correlation because of the categorical nature of the item responses, is 0.92.

Impulsivity traits were measured with the short version of the UPPS-P Impulsive Behavior Scale [47]. The UPPS-P is a self-reported questionnaire that assesses five distinct impulsive traits: negative urgency (e.g., “When I am upset I often act without thinking”; Cronbach’s alpha for the current sample: 0.82), positive urgency (e.g., “When I am really excited, I tend not to think of the consequences of my actions”; Cronbach’s alpha for the current sample: 0.75), lack of perseverance (e.g., “I finish what I start”; Cronbach’s alpha for the current sample: 0.86), lack of premeditation (e.g., “Before making up my mind, I consider all the advantages and disadvantages”; Cronbach’s alpha for the current sample: 0.82), and sensation seeking (e.g., “I sometimes like doing things that are a bit frightening”; Cronbach’s alpha for the current sample: 0.84). Previous research conducted with the UPPS-P was characterized as having a solid factorial structure, adequate test-retest validity, and high internal reliability [47].

### Data analyses

Descriptive statistics about FB use and preferences, along with gender comparisons, are presented in Tables 1 and 2. Chi-square tests were computed for FB use, whereas the Wilcoxon test was used for age and rank variables, since the distributions were skewed.

Regression analyses were computed to identify predictors of problematic FB use. In all regression models computed, the dependent variable was the total score on the IAT-R and the independent variables entered were sex, age, FB activities, and impulsivity traits (UPPS-P). A hierarchical regression strategy was performed. In a first step, only sex, age and FB activities were entered in the model. In a second step, impulsivity subscales (UPPS-P) were entered. Residual analysis showed problems such as non-normality and outliers; therefore, the logarithm of the IAT score was used instead and this transformation provided satisfactory residual analysis. From the variance inflation factors (VIF), no sign of multicollinearity was found.

All statistical analyses were done with R 3.3.0 [48]). All study data are available from https://osf.io/xt4zv/.

## Results

**Fig 1:**
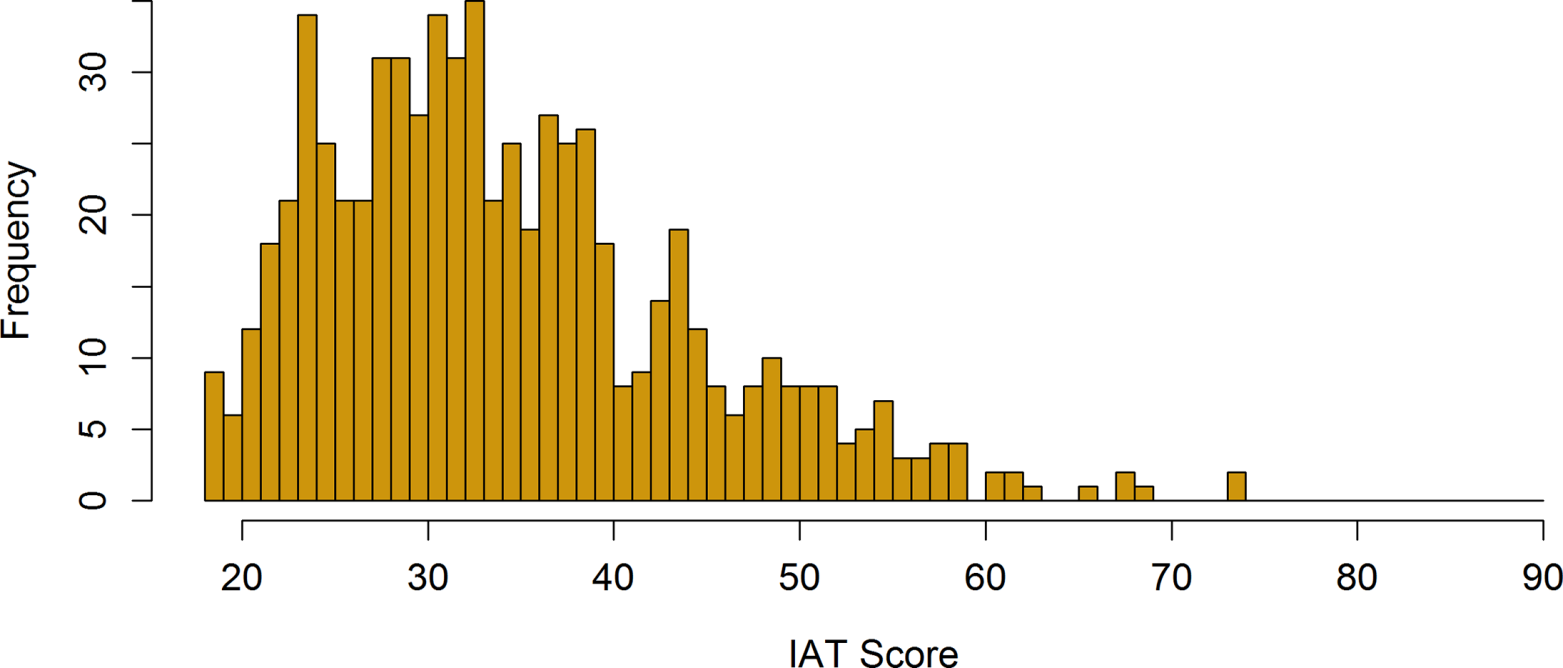
Distribution of the IAT scores. Theoretical score range: 18–90; Actual score range: 18–74.

Fig 1 shows the distribution of the IAT scores. The theoretical scores range is between 18 and 90 while the actual scores range is between 18 and 74. 25% of the participants had a score lower that 28, the median score was 33 and 75% of the subjects had a score lower than 40.

The first part of Table 1 shows the types of activities performed on FB. The most prevalent FB-related activity appeared to be reading the news feed, followed next by viewing friends’ pictures. Gender differences appeared, however, among the types of activities performed on FB. Indeed, reading the news feed was the most often reported activity by men, whereas it was the second-most often reported activity for women, for whom viewing friends’ pictures was the most prevalent activity. Notably, gaming was the seventh most prevalent reason to use FB, which was the penultimate one (the last for men). Statistically significant differences occurred between genders, the first one being reading the news feed, which men reported doing more often than women did (65.2% vs. 54.1%). In addition, twice as many men update their status compared to women (45.2% vs. 28.8%), whereas gaming had the reverse pattern (11.6% of women reported gaming on FB vs 5.7% of men). The second part of Table 1 shows the type of FB access, with 43.5% of respondents declaring that they use the Messenger app and 44.7% that they use notifications. Women reported using notifications (48.7%) more often than men did (35.7%). Most of the subjects (87.6%) reported using FB mainly on their personal computer or their smartphone, but there was a gender difference for main access, as men preferred to use their personal computer (47.1%) and women preferred to use their smartphone (57.9%).

Table 2 reports ranked preferred activities performed on FB. Although reading the news feed had the smallest rank for both men and women, men notably ranked gaming as the second activity, whereas women ranked reading friends’ timelines as second. Moreover, the mean rank for gaming was statistically lower for men (1.9) than for women (3.1), whereas updating their status was higher for women (2.8) than for men (2.2).

Table 3 shows the results of the hierarchical regression analysis computed to predict problematic use of FB (logarithm of the total score on the IAT-R). In a first step, only gender, age, FB activities, and use were entered in the model. Age was negatively associated with problematic FB use, whereas both updating one’s status and gaming were positively associated with it. Interestingly, the use of notifications was also positively associated with problematic use of FB. In a second step, impulsivity subscales were added to the model. The increase in the *R*-squared value between Model 1 and Model 2 was statistically significant. All the previous statistically significant associations from the first model remained significant in the second model, except for notifications. Notifications were nevertheless barely not significant, which may be due to a decrease in power or impulsivity that could have had a mediating effect because negative urgency, positive urgency, and lack of premeditation were positively associated with problematic FB use. The *R*-squared value of Model 2 was more than twice that of Model 1 and was statistically significant, suggesting that adding impulsivity facets dramatically increased the quality of the model.

**Table 3:** Predictors of FB problematic use: Hierarchical regressions.

	Model 1				Model 2			
	Estimate	*SE*^*1*^	*t*	*p*-Value^2^	Estimate	*SE*	*t*	*p*-Value^2^
**Women vs. Men**	-0.039	0.024	-1.66	0.10	-0.023	0.023	-1.01	0.31
**Age**	-0.003	0.001	-3.29	**>0.01**	-0.003	0.001	-2.82	**>0.01**
**FB activities (yes vs. no):**								
***Updating status***	0.090	0.024	3.76	**>0.001**	0.090	0.022	4.03	**>0.001**
***Reading the news feed***	0.035	0.022	1.58	0.11	0.018	0.021	0.87	0.39
***Reading friends’ timelines***	0.012	0.022	0.57	0.57	0.002	0.020	0.09	0.92
***Commenting***	-0.019	0.024	-0.81	0.42	-0.030	0.022	-1.37	0.17
***Viewing friends’ pictures***	0.014	0.022	0.63	0.53	0.009	0.021	0.44	0.66
***Gaming***	0.096	0.036	2.68	**>0.01**	0.079	0.034	2.34	**0.02**
***Contributing to a group***	0.040	0.023	1.77	0.08	0.034	0.021	1.64	0.10
***Sharing stuff from the Internet***	-0.013	0.042	-0.30	0.77	-0.025	0.040	-0.63	0.53
**Messenger app (yes vs. no)**	0.014	0.023	0.61	0.54	0.010	0.022	0.48	0.63
**FB main access (vs. personal computer):**								
***Professional computer***	-0.037	0.043	-0.85	0.40	-0.052	0.041	-1.27	0.20
***Smartphone***	-0.039	0.024	-1.59	0.11	-0.044	0.023	-1.93	0.05
***Tablet***	-0.070	0.048	-1.48	0.14	-0.072	0.044	-1.63	0.10
**Notifications (yes vs. no)**	0.049	0.023	2.15	**0.03**	0.039	0.021	1.85	0.06
***Impulsivity traits*:**								
***Negative urgency***					0.009	0.005	2.03	**0.04**
***Positive urgency***					0.022	0.005	4.15	**>0.001**
***Lack of premeditation***					-0.006	0.005	-1.07	0.29
***Lack of perseverance***					0.032	0.005	6.53	>0.001
***Sensation seeking***					0.001	0.004	0.37	0.72
***R*^2^**	0.075				0.206			
**Delta *R*^2^**					0.130			**>0.001**

^1^Standard error of the regression coefficient.

^2^p-Values smaller than 0.05 are in bold.

## Discussion

In the current study, we first aimed to highlight how FB use is multidetermined and related to a range of specific activities, and we second sought to identify which types of usage preferences and impulsivity profiles predict problematic involvement in FB. On the whole, we identified that various motives and related activities “fuel” FB use and that gender differences exist in relation to FB usage preferences. Specific usage preferences (i.e., updating status, gaming via FB, and using notifications) and impulsive personality traits (i.e., positive urgency, negative urgency, lack of perseverance) are related to problematic use of FB. Similar to what has been found in previous studies, younger age was also associated with increased excessive use of FB [10].

The current study first provides, in a sample of French-speaking individuals, insight into FB usage preferences (see Tables 1 and 2 for related results) and their potential predictive value in explaining symptoms of problematic use. Among the various results related to FB usage preferences, three specific findings are of much importance and deserve further elaboration. The first is that the tendency to update FB status is an important predictor of problematic use of FB. A possible explanation for this finding is that constantly updating one’s status probably interferes with daily activities and thus engenders negative consequences. For example, proneness to frequently update one’s status likely negatively impacts on professional or academic productivity through the frequent switching of one’s focus of attention from the activity in question to FB. Proneness to frequently update one’s status is also likely to have negative social consequences. This will particularly be the case when smartphone users update their status in social situations, resulting in “phubbing” behaviors, i.e., the act of snubbing someone in a social setting by using one’s phone instead of interacting [49]. A second important finding pertains to the fact that gaming through FB is an important predictor of problematic usage. In this regard, it is possible to assert that gaming is probably a highly addictive SNS-mediated activity. Such a result supports the view that labels such as “FB addiction” or “SNS addiction” are misleading and that a focus on the actual activities performed on SNSs is crucial when making inferences regarding potential dysfunctional usage. Such an argument is in line with recent criticisms made about other umbrella constructs such as “Internet addiction” [50] or “mobile phone addiction” [51]. In relation to FB-mediated gaming, our study also highlighted that there are a higher proportion of female gamers, which may seem at first sight surprising. Nevertheless, a possible explanation is the nature of video games that are generally played via FB, which mainly belong to the category of “casual games” (prototypical examples: Candy Crush Saga, Farmville). These video games, which are generally easy to handle and can be played for even short sessions of a few minutes are more popular among females [52], which could explain the gender difference observed in our study. We highlighted that using notifications is a predictor of problematic FB use. Even if this result becomes marginally significant (*p* = .06) when impulsivity-related variables are included in the regression model, it suggests that switching off FB-related notifications can constitute a protective factor against problematic use.

The current research report also showed that specific impulsivity traits predict problematic involvement in FB. First, our results emphasized that problematic use of FB is predicted by heightened positive and negative urgency. Accordingly, disordered FB use is linked to emotion-laden impulsive behaviors and can thus be conceptualized as a dysfunctional strategy to cope with aversive emotions (for individuals with high negative urgency) or a way to promote or maintain pleasant emotions (for individuals with high positive urgency), despite the potential resulting negative consequences (e.g., conflicts, interference with daily life routines, reduced sleep). In other words, individuals with a high (positive and/or negative) urgency trait are at increased risk of developing FB usage that constitutes a maladaptive emotion regulation strategy associated with tangible negative consequences. Such an explanation is in line with the large body of evidence suggesting that a wide range of problematic behaviors, such as alcohol abuse [53] and compulsive buying [54], can be viewed as urgency-related behaviors displayed to regulate (suppress and/or exacerbate) emotional states in the short term despite their delayed negative consequences [36,55,56]. Of importance, heightened urgency has also been related to reduced inhibitory control and poor decision making [57,58], implying that the deregulated use of FB demonstrated by individuals with a high urgency trait could be sustained by conjoint deficits in emotion regulation and executive control skills. Yet, the few preliminary data available from the literature tend to emphasize persevered frontal lobe functioning in individuals displaying excessive FB use [59,60], implying that further research is needed to better understand the psychological and neurological processes at play. Second, the lack of a perseverance facet of impulsivity was also found to predict addictive use of FB. This impulsivity component, defined as difficulty in remaining focused on cognitively demanding and/or boring tasks [37], has been related to attentional difficulties [61] and to an increased occurrence of distractions or irrelevant thoughts that may interfere with ongoing tasks or project completion [62,63]. Lack of perseverance, among other things, was found to predict procrastination behaviors [64] and elevated frequency of mobile phone use [65]. Accordingly, we postulate that a lack of perseverance increases actual and potentially exaggerated use of FB because of attentional fluctuation, mind wandering, or irrelevant thoughts (e.g., someone checking a friend’s page following an intrusive thought about this friend; someone checking FB following difficulties in concentrating on an ongoing task). Such an assumption is in line with previous results that linked problematic use of SNSs with symptoms of attention-deficit hyperactivity disorder [10]. Notably, because of the correlational design of the study, we cannot exclude a reverse explanation, i.e., problematic use of FB in itself interferes with other competing tasks and thus promotes lack of perseverance.

Some limitations of the study warrant consideration. First, the sample is self-selected and thus not necessarily representative of the population under study. For example, according to Sprout Social (https://sproutsocial.com/insights/new-social-media-demographics/), 44% of FB users are females (at the worldwide level), implying that they are overrepresented in our sample. Although not problematic in relation to our findings regarding the factors involved in problematic FB use, this nonetheless calls for extra caution when considering the usage patterns and preferences as potentially reflecting those of the FB French-speaking community. Second, the questions related to FB use and preferences were generated in the framework of the current study, implying that we cannot exclude the possibility that additional reasons or motives to use FB would have been identified with other methodologies (e.g., focus groups, open-ended questions, usage time trackers). Third, the analyses regarding gaming were drawn from a rather small sample (12 men and 54 women), therefore the results should be taken with caution. Finally, the current study relied on self-reports, which makes the data collected prone to be influenced by lack of insight and social desirability biases [66].

Despite these limitations, the current study offers several original results that could fuel further research. Indeed, given the heterogeneous nature of SNS-related activities, future studies should systematically measure the activities performed, rather than considering the SNS user population as a homogenous group. As an illustration, our results suggest that individuals gaming on FB are more prone to display symptoms of problematic use, which could imply that some video games playable via FB are addictive, but not necessarily that FB is addictive per se. At a broader level, the links observed between various impulsivity traits and problematic FB use are in accordance with those presented in recent theoretical models [35,67], which posit that impulsivity and related mechanisms (e.g., impairment in inhibitory control and decision making) play a pivotal role in the onset and maintenance of specific Internet use disorders. At a more applied level, the finding that using notifications predicts addictive-like use of FB has straightforward implications in terms of prevention of problematic SNSs use. Furthermore, it is reasonable to suppose that a similar approach can be generalized to other types of notifications (e.g., most smartphone apps send notifications) and thus to a wide range of potentially problematic online applications.
